# Profil épidémiologique, clinique et thérapeutique des atrésies de l’œsophage au Centre Hospitalier Universitaire Mère-Enfant Fondation Jeanne Ebori de Libreville de 2019 à 2020

**DOI:** 10.11604/pamj.2023.44.34.30880

**Published:** 2023-01-18

**Authors:** Melina Nkole Aboughe Obame, Aude Lembet Mikolo, Bobby Nguele Ndjota, Emmanuel Comlan, Marcelle Abegue, Francois Ondo Ndong

**Affiliations:** 1 Service de Chirurgie Pédiatrique, Centre Hospitalier Universitaire Mère-Enfant Fondation Jeanne Ebori, Libreville, Gabon,; 2 Pôle Pédiatrie, Centre Hospitalier Universitaire Mère-Enfant Fondation Jeanne Ebori, Libreville, Gabon,; 3 Département de Chirurgie, Université des Sciences de la Santé, Libreville, Gabon,; 4 Service de Médecine Néonatale, Centre Hospitalier Universitaire Mère-Enfant Fondation Jeanne Ebori, Libreville, Gabon,; 5 Département de Pédiatrie, Université des Sciences de la Santé, Libreville, Gabon,; 6 Service de Chirurgie du Centre Hospitalo-Universitaire de Libreville, Libreville, Gabon,; 7 Service de Réanimation, Centre Hospitalier Universitaire Mère-Enfant Fondation Jeanne Ebori, Libreville, Gabon

**Keywords:** Atrésie de l’œsophage, Libreville, urgence chirurgicale, prise en charge, Esophageal atresia, Libreville, surgical emergency, management

## Abstract

L´atrésie de l´œsophage est une malformation congénitale incompatible avec la vie. Sa prise en charge dans notre contexte reste difficile. Notre étude a pour but de déterminer le profil épidémiologique, clinique et thérapeutique des atrésies de l´œsophage au Centre Hospitalier Universitaire Mère-Enfant Fondation Jeanne Ebori de Libreville de 2019 à 2020. Nous avons réalisé une étude rétrospective sur 2 ans allant du 1^er^ janvier 2019 au 31 décembre 2020 dans les services de chirurgie pédiatrique et de médecine néonatale du Centre Hospitalier Universitaire Mère-Enfant Fondation Jeanne Ebori. Nous avons colligé 10 nouveau-nés diagnostiqués pour atrésie de l´œsophage. La prévalence est de 11% des urgences chirurgicales néonatales (n=10/89). L´âge gestationnel moyen était 37SA+2 jours avec des extrêmes 34SA+2 jours et 40SA+2 jours. L´âge de vie moyen était de 3,7 jours avec des extrêmes de J0 et J7. Le sex-ratio était de 1. Le poids moyen était de 2636 grammes avec des extrêmes de 1460 g et 3425g. Le délai moyen du diagnostic était de 4 jours. Le type III représentait 70% des formes anatomiques. Le délai moyen de prise en charge chirurgicale était de 0,8 jours avec des extrêmes de J0 et J3. La durée moyenne d´hospitalisation était de 9,3 jours avec des extrêmes 1 et 38 jours. Neuf patients sur 10 ont été opérés. Seulement 2 patients ont survécu avec un recul d´au moins un an sans séquelles. La prise en charge de l´atrésie de l´œsophage reste encore précaire dans notre contexte. La première difficulté est le retard diagnostic. L´amélioration de son pronostic passe par l´amélioration du plateau technique anesthésique, chirurgical et néonatal.

## Abstract (en)

Esophageal atresia is a congenital malformation incompatible with life. Its management in our context is difficult. The purpose of this study was to determine the clinical, therapeutic and epidemiological profile of esophageal atresia at the Mother and Child University Hospital of the Jeanne Ebori Foundation from 2019 to 2020. We conducted a retrospective study in the Neonatal Medicine and Pediatric Surgery Department of Mother and Child University Hospital of the Jeanne Ebori Foundation over a 2-year period from January 1, 2019 to December 31, 2020. We collected data from the medical records of 10 newborns diagnosed with esophageal atresia. The prevalence of neonatal surgical emergencies was 11% (n=10/89). Mean gestational age of patients was 37 WA+2, ranging from 34WA+2 days to 40WA+2 days; mean life span was 3.7 days with extremes of D0 and D7. Sex ratio was 1. The average birth weight was 2636 grams, ranging from 1460 g to 3425g; mean time between symptom onset and diagnosis was 4 days. Type III accounted for 70% of anatomical shapes. Mean waiting time for surgery was 0.8 days, ranging from D0 to D3. The average length of stay in hospital was 9.3 days, ranging from 1 to 38 days. Nine out of 10 patients underwent surgery. Only 2 patients survived, with a follow-up period of at least one year without sequelae. The management of esophageal atresia is still precarious in our context. Diagnostic delay is the main challenge. Improvement in prognosis involves improvement in anesthetic, surgical and neonatal technical equipment.

## Introduction

L´atrésie de l´œsophage est une malformation congénitale qui correspond à une interruption de la continuité de l´œsophage. Il en découle non seulement une impossibilité à s´alimenter mais aussi une inondation trachéobronchique. Il s´agit donc d´une extrême urgence chirurgicale. C´est une malformation congénitale rare (1 sur 2 500 à 3 500 naissances), dont le pronostic actuel est bon [1] pour ce qui est des pays développés. Sa prise en charge dans notre contexte reste difficile du fait du plateau technique limité et, elle est grevée d´une forte mortalité. Notre étude a pour objectif général de déterminer le profil épidémiologique, clinique et thérapeutique des atrésies de l´œsophage au Centre Hospitalier Universitaire Mère-Enfant Fondation Jeanne Ebori (CHUMEFJE). Les objectifs spécifiques pour atteindre cela sont: de calculer la prévalence des atrésies de l´œsophage, de donner les caractéristiques socio-démographiques, d´évaluer les délais diagnostiques et thérapeutiques, enfin de déterminer la mortalité de cette affection.

## Méthodes

**Type d´étude:** nous avons réalisé une étude rétrospective et descriptive.

**Cadre et population de l´étude:** l´étude s´est déroulée dans les services de médecine néonatale et de chirurgie pédiatrique du CHU Mère-Enfant Fondation Jeanne Ebori durant une période de 2 ans allant du 1^er^ janvier 2019 au 31 décembre 2020. Il s´agit d´une structure sanitaire de niveau 3 située dans la capitale politique du Gabon. Durant cette période, nous avons inclus, tous les nouveau-nés ayant présenté une atrésie de l´œsophage.

**Les paramètres étudiés chez le nouveau-né étaient:** l´âge gestationnel (SA), le poids de naissance, le sexe, le délai diagnostic, le délai de prise en charge, le traitement chirurgical, la durée d´hospitalisation et l´évolution.

**Analyse des données:** les données ont été recueillies à partir des dossiers médicaux des patients et des registres du bloc opératoire. Elles ont été saisies et analysées sur Excel 2013.

## Résultats

Au cours de la période d´étude, 89 nouveau-nés ont été opérés pour une urgence chirurgicale au Centre Hospitalier Universitaire Mère-Enfant Fondation Jeanne Ebori (CHUMEFJE). Le nombre de nouveau-nés ayant présenté une atrésie de l´œsophage était de 10 soit une prévalence de 11%. Le poids moyen était de 2636 grammes et l´âge gestationnel moyen était de 37 SA+2 jours (Tableau 1). L´âge maternel moyen était de 23,6 ans avec des extrêmes de 21 et 34 ans. Toutes les grossesses étaient suivies. Seulement 2 échographies ont mis en évidence un hydramnios. Neuf patientes sur dix étaient de nationalité gabonaise. Au total 8/10 patients étaient des patients outborn (5 proviennent de structures privées, 3 CHU dont 2 CHUME et 1 CHUO, 1 centre de santé, 1 CHR). L´âge moyen d´admission était de 3,7 jours avec des extrêmes J0 et J7. Le diagnostic a été posé selon les données cliniques et para cliniques étayées dans le Tableau 2. Ce dernier met en évidence le fait que tous les patients avaient pour signe évocateur de la pathologie une détresse respiratoire et une hypersialorrhée. Les différentes malformations associées retrouvées sont représentées dans le Tableau 3. La plupart d´entre elles s´avérait être soit d´origine cardiaque, soit des malformations ano-rectales.

**Tableau 1:** paramètres du nouveau-né

		Effectif (n)	Pourcentage (%)
**Age gestationnel (SA)**	Terme	8	80
	Prématurité moyenne	2	20
**Poids de naissance (g)**	< 1500	1	10
	1500-2500	3	20
	> 2500	7	30
**Sexe (Sex-ratio=1)**	Masculin	5	50
	Féminin	5	50

**Tableau 2:** signes cliniques

Signes	Nombre (n)	Pourcentage (%)
Détresse respiratoire	10	100
hypersialorrhée	10	100
Butée de la sonde gastrique	10	100
Malformations associées	6	60
Aération digestive	8	80

**Tableau 3:** malformations associées

Malformations	Effectif
CIA+CIV	1
CIV	1
MAR	1
MAR+malformation oreille externe+HIG+sténose VP	1
MAR+HTA+malformations vertébrales	1
HIB	1

La forme anatomique la plus retrouvée était le type III (7/10), elle était suivie du type I dans 2 cas et du type 5 retrouvé chez un patient. Neuf (9) patients sur 10 ont été opérés. Ils ont bénéficié d´une gastrostomie dans 2 cas, d´une oesophagoplastie couplée à une colostomie chez 2 patients et enfin 5 patients ont bénéficié d´une oesophagoplastie par anastomose termino-terminale. Sur le plan médical, les patients ont été mis sous-alimentation parentérale (acides aminés) dès J1. Ils bénéficiaient d´une antibiothérapie de première ligne (céfotaxime+ gentamycyne) en pré opératoire; en post opératoire immédiat l´antibothérapie étaient d´emblée de seconde ligne (imipénème +vancomycine); enfin dans les cas de surinfection une antibiothérapie de troisième ligne était administrée (ciprofloxacine seul ou en association avec méropénème). Le délai diagnostic moyen était de 4 jours avec des extrêmes de 0 et 7 jours. Le délai d´intervention moyen était de 0,8 jours avec des extrêmes de J0 et J3. La durée moyenne d´hospitalisation était de 9,3 jours avec des extrêmes de 1 et 38 jours. La mortalité globale était de 80% (Tableau 4).

**Tableau 4:** évolution

	Effectif (n)	Pourcentage %
Nombre de patients vivants	2	20
Nombre de décès post-opératoire	7	70
Nombre de décès pré-opératoire	1	10
Nombre total de décès	8	80

## Discussion

Les atrésies de l´œsophage représentaient 11% des pathologies admises en médecine néonatale durant notre période d´étude. Le sexe ne semble avoir aucune incidence sur la survenue de cette pathologie. Ils avaient une moyenne d´âge de 37SA+2 jours. Les délais diagnostiques étaient de 4 jours et les délais thérapeutiques de 0,8 jours en moyenne. Notre étude était limitée par le fait qu´elle était rétrospective, nous ne pouvions tenir compte que des données recueillies sur les dossiers. L´atrésie de l´œsophage est une urgence dont le pronostic dépend de la précocité de la prise en charge et donc du diagnostic. Dans les pays développés le diagnostic anténatal est possible et réalisé au moins une fois sur deux [1] . Nous avons retrouvé 2 échographies ayant révélé un hydramnios en anténatal dans notre cohorte. Ehua *et al*. [2] en Côte d´Ivoire aussi avaient retrouvé un hydramnios chez deux sur huit de ses patients.

Mouafo *et al*. [3] au Cameroun et Mbaye *et al*. [4] au Sénégal n´ont bénéficié d´aucun élément évocateur de diagnostic en période anténatale. Cette affection étant méconnue n’est donc pas systématiquement recherchée en anténatal (hydramnios, pouch sign à l´échographie) ni même en salle d´accouchement ou la systématisation de l´épreuve de la sonde naso-gastrique autrefois de rigueur semble avoir reculé. Ceci explique le délai diagnostic tardif de 4 jours qui est le nôtre. Il est sensiblement le même chez nos voisins du Cameroun [3]: 3,5 jours mais bien plus tardif à Treichville [2] soit 6,6 jours. Pourtant l´ensemble de ces accouchements ont eu lieu en milieu hospitalier (l´un d´entre eux a été réalisé dans un centre de santé mais cela reste une structure sanitaire).

Nous avons tout de même constaté que 8 des dix patients étaient nés hors de notre structure. Selon Mohamed B [5], 74% de leurs patients étaient outborns. Le sexe ne semble avoir aucune incidence sur la pathologie, il en est de même pour l´équipe de Bandre [6]. Le type d´atrésie de l´œsophage le plus fréquemment retrouvé était le type III (70%). Cela concorde avec les données de la littérature. Bouguermouh *et al*. [7] et Ben Kirane *et al*. [5] trouvaient respectivement 67% et 74% de type III. Nous avons réalisé un bilan malformatif qui a objectivé une prédominance pour les malformations cardiaques suivies des malformations ano-rectales. Al-Salam *et al*. [8] en Arabie Saoudite ont retrouvé 50% de malformations associées dont 49% d´origine cardiaque; Tandon *et al*. [9] en Inde aussi ont constaté ces mêmes comorbidités. Harifetra *et al*. [10] à Madagascar n´ont pas réalisé de bilan malformatif du fait de son indisponibilité. L´atrésie de l´œsophage est incompatible avec la vie à moins d´être prise en charge chirurgicalement dans des délais convenables. Elle est de facto rencontrée chez les sujets fragiles que sont les nouveau-nés. Ils sont de faibles poids, en moyenne 2600 grammes, frappés en majorité de malformations associées notamment cardiaque.

Cela représente un défi non seulement pour le chirurgien mais aussi l´anesthésiste et le réanimateur néonatal. Ces comorbidités assombrissent lourdement le pronostic. Les 2 patients ayant présenté une atrésie long gap de type I ont bénéficié d´une gastrostomie d´attente. L´équipe d´Arabie Saoudite [8] elle aussi procédait à des gastrostomies dans les atrésies ou la distance entre les culs de sac était longue. Nous avons procédé avec le reste de nos patients à des thoracotomies droites extra pleurales lorsque cela était possible. Tandon *et al* [9] ont aussi eu recours à cette approche thérapeutique pour la majorité de leurs patients. Le délai moyen d´intervention dans notre structure était de 0,8 jours. Les délais de Mouafo [3] et Mbaye [4] étaient respectivement de 4,8 jours et 5,7 jours. Le CHUMEFJE a l´avantage de rassembler en son sein tous les chirurgiens pédiatres du pays, un service de néonatalogie, une équipe d´anesthésistes réanimateurs ainsi que le laboratoire et la radiologie disponibles 24h/24. Malheureusement malgré la précocité de notre prise en charge chirurgicale l´évolution est tout de même sanctionnée par le décès de 8 patients sur 10. Mouafo en a perdu 9 sur 10 au Cameroun et Randriamizao 14 sur 17 à Madagascar [10]. La survie de 2 de nos patients avec un recul d´un an montre qu´il est possible de prendre en charge avec succès cette affection dans notre pays.

## Conclusion

Nous nous étions fixés pour objectif général de déterminer le profil épidémiologique, clinique et thérapeutique des atrésies de l´œsophage au Centre Hospitalier Universitaire Mère-Enfant Fondation Jeanne Ebori de Libreville. Les atrésies de l´œsophage représentaient 11% des urgences chirurgicales admises en médecine néonatale durant notre période d´étude. Le sexe ne semble avoir aucune incidence sur la survenue de cette pathologie. Ils avaient une moyenne d´âge de 37SA+2 jours. Les délais diagnostiques étaient de 4 jours et les délais thérapeutiques de 0,8 jours en moyenne. Elle est une hantise pour le chirurgien pédiatre dans les pays en voie de développement, car sa mortalité reste élevée. La première difficulté rencontrée est le retard diagnostic. L´amélioration de son pronostic passe par l´amélioration du plateau technique médical, paramédical instrumental ainsi que de la collaboration entre les gynécologues obstétriciens, les pédiatres et chirurgiens pédiatres.

### Etat des connaissances sur le sujet

- *L´atrésie de l´œsophage est une urgence chirurgicale qui correspond à une interruption de la continuité de l´œsophage;*
- *Son diagnostic est souvent anténatal en Europe, tandis qu´en Afrique sub-saharienne il est peu ou pas réalisé;*
- *Elle est grevée d´une forte mortalité dans les pays en voie de développement contrairement au pays du Nord*.

### Contribution de notre étude à la connaissance

- *Notre étude rapporte l´expérience de l´unique service nationale de chirurgie pédiatrique au Gabon;*
- *Il s´agit d´une étude préliminaire réalisée devant la pauvreté de données sur la question dans notre pays;*
- *La faible survie retrouvée démontre toutefois qu´il est possible de traiter cette affection dans notre contexte*.
